# Reinforcement learning in motor skill acquisition: using the reward positivity to understand the mechanisms underlying short- and long-term behavior adaptation

**DOI:** 10.3389/fnbeh.2024.1466970

**Published:** 2024-10-30

**Authors:** Mariane F. B. Bacelar, Keith R. Lohse, Juliana O. Parma, Matthew W. Miller

**Affiliations:** ^1^Department of Kinesiology, Boise State University, Boise, ID, United States; ^2^Program in Physical Therapy, Washington University School of Medicine, St. Louis, MO, United States; ^3^Department of Kinesiology, San Francisco State University, San Francisco, CA, United States; ^4^School of Kinesiology, Auburn University, Auburn, AL, United States; ^5^Center for Neuroscience Initiative, Auburn University, Auburn, AL, United States

**Keywords:** motor learning, reward-prediction errors, graded feedback, EEG, mixed-effects modeling

## Abstract

**Introduction:**

According to reinforcement learning, humans adjust their behavior based on the difference between actual and anticipated outcomes (i.e., prediction error) with the main goal of maximizing rewards through their actions. Despite offering a strong theoretical framework to understand how we acquire motor skills, very few studies have investigated reinforcement learning predictions and its underlying mechanisms in motor skill acquisition.

**Methods:**

In the present study, we explored a 134-person dataset consisting of learners’ feedback-evoked brain activity (reward positivity; RewP) and motor accuracy during the practice phase and delayed retention test to investigate whether these variables interacted according to reinforcement learning predictions.

**Results:**

Results showed a non-linear relationship between RewP and trial accuracy, which was moderated by the learners’ performance level. Specifically, high-performing learners were more sensitive to violations in reward expectations compared to low-performing learners, likely because they developed a stronger representation of the skill and were able to rely on more stable outcome predictions. Furthermore, contrary to our prediction, the average RewP during acquisition did not predict performance on the delayed retention test.

**Discussion:**

Together, these findings support the use of reinforcement learning models to understand short-term behavior adaptation and highlight the complexity of the motor skill consolidation process, which would benefit from a multi-mechanistic approach to further our understanding of this phenomenon.

## Introduction

The process of acquiring motor skills is typically marked by rapid improvements in performance observed early in practice followed by smaller adjustments when the learner achieves a higher skill level (e.g., power law of practice; Newell and Rosenbloom, 1981). Given the complexity of the elements involved in this process, different modes of learning have been jointly used to explain how we acquire motor skills (Lohse et al., 2019; Seidler et al., 2013; Wolpert et al., 2011). For instance, error-based learning asserts that trial-to-trial adjustments in performance are guided by the discrepancy between the expected and actual sensory consequences of a motor command (i.e., a sensory-prediction error). This type of learning is thought to engage cerebellar-cortical pathways (McNamee and Wolpert, 2019; Wolpert et al., 1998) and rely on forward models (Diedrichsen et al., 2010) to generate behavioral adjustments. Specifically, based on the current state of the motor system and the action to be executed, the internal forward model anticipates the sensory feedback, which is contrasted against the actual sensory feedback (Haith and Krakauer, 2013a). Early in practice, this discrepancy is large, likely due to the lack of a reliable and accurate forward model of the skill (Wolpert et al., 1998). Thus, larger motor command adjustments are implemented in an attempt to reduce the sensory-prediction error. As performance improves, the internal representation of the skill becomes more accurate and the discrepancy between planned and actual sensorimotor outcome diminishes, which culminates in smaller motor command adjustments.

Whereas error-based learning explains movement refinement on the account of sensory-reward prediction errors, reinforcement learning, another important model to understand skill acquisition, explains performance adjustments based on *reward-prediction errors*, the difference between actual and anticipated rewards (Rescorla, 1972; Schultz, 2017; Sutton and Barto, 1998). Behaviors that lead to better- or worse-than-expected outcomes result in positive and negative reward-prediction errors, respectively. At the neural level, reward-prediction errors convey information that is used to guide future adaptations (Seidler et al., 2013). More specifically, within the brain, positive reward-prediction errors increase the value of behaviors that resulted in better-than-expected outcomes, making the re-occurrence of these behaviors more likely in the future. Conversely, negative reward-prediction errors decrease the value of behaviors that resulted in worse-than-expected outcomes, making the re-occurrence of these behaviors less likely in the future. Consider the practical example of a novice trying to learn how to putt. Early on, her lack of familiarity with the task and ability to detect and correct errors may lead to frequent, large negative reward-prediction errors due to her badly missed putts. Thus, to find the movement pattern that will get her closer to sinking a putt, she needs to explore different movement strategies (i.e., implement large performance adjustments). Her lack of practice and experience also makes successful performance (i.e., sinking the putt) less likely to occur, so her expectations for future rewards are low. Thus, when she unexpectedly sinks her first putt, this leads to an outcome that is far better than anticipated or in other words, a large positive reward-prediction error. As previously mentioned, positive reward-prediction errors facilitate movement repetition so the behavior that precipitated success is likely to be repeated, leading to rapid improvements. Toward the later stages of learning, she may have already found the movement strategy that more closely aligns with the optimal movement pattern. At this point, she begins to exploit that movement strategy to find *her* optimal movement pattern by implementing smaller adjustments. Also, as she becomes more skillful and knowledgeable about the task, her actual performance starts to match her expected performance, leading to smaller reward-prediction errors, which would explain the smaller performance adjustments seen at that stage.

Under an error-based learning framework, once motor errors are on average reduced to zero, further improvements are limited. Due to motor control redundancy (i.e., different combinations of movement adjustments can lead to the same end result; Haith and Krakauer, 2013b), the exploration of the motor solution space to find the *optimal* movement requires the involvement of other learning mechanisms, such as reinforcement learning (Wolpert et al., 2011). Thus, investigating how these systems use different learning signals (sensory errors vs. outcome success/failure) and work together to promote behavior adaptation is crucial to enhance our understanding of the motor skill acquisition process. Although both error-based learning and reinforcement learning have been well studied across different paradigms, most of these investigations have focused on motor adaptation (e.g., Diedrichsen et al., 2010; Izawa and Shadmehr, 2011; Pélisson et al., 2010). Thus, it is still unclear how reinforcement learning mechanisms contribute to what behavioral researchers define as motor skill *learning*; i.e., a relatively permanent change in the capability for behavior, not merely an adjustment made on the next attempt (Kantak and Winstein, 2012; Schmidt and Lee, 2019). To expand our understanding of this relationship, in the present study, we focus on the application of reinforcement learning to explain motor skill acquisition by adopting a mechanistic approach to investigate one of its main drivers, reward-prediction errors.

In human research, reward-prediction errors have been studied through the measure of the reward positivity (RewP), an event-related potential (ERP) component derived from the electroencephalogram (EEG). Methodologically, the RewP is characterized as a positive deflection in the ERP waveform that peaks between 230 and 350 ms after augmented feedback onset and exhibits a frontal-central scalp topography, typically maximal at electrode FCz (Krigolson, 2018). While support for the association between RewP and behavior exists (Holroyd and Krigolson, 2007; Williams et al., 2018), studies investigating this relationship often do not use learnable tasks, relying instead on those where performance and feedback are based on chance (e.g., reward gambling tasks). In these paradigms the task is typically simple, and feedback is usually binary (i.e., squeeze a dynamometer and receive correct versus incorrect feedback response; Meadows et al., 2016). Moreover, the frequency and/or probability of receiving correct/incorrect feedback is controlled by the experimenter (e.g., probability of making a correct response and receiving positive feedback is set at 50%). However, real-world skill acquisition involves more complex, learnable tasks wherein feedback probability varies as a function of performance. Additionally, sensory information is available to error-based learning systems and is supplemented by graded outcome-based feedback (e.g., coach: “you overshot the target by 35 cm”). Augmented feedback as defined by information that is fed back to the learner via artificial means (e.g., a coach providing verbal feedback; Schmidt and Lee, 2019) plays a major role in performance improvement (Schmidt and Lee, 2019), especially at the earlier stages of learning (Newell, 1976), and from a motor learning perspective, graded feedback is more advantageous as it provides learners with more information that can be used to flexibly make performance adjustments.

Very few studies have investigated the relationship between RewP and graded feedback processing (e.g., Ulrich and Hewig, 2014), and fewer have done so using a motor learning paradigm. One exception is the study by Frömer et al. (2016) in which participants performed a virtual throwing task and received visual graded feedback about where each throw landed relative to the target’s bullseye. Results from this study showed that more accurate throws resulted in larger RewP amplitudes, which is in line with the reinforcement learning prediction that larger rewards (i.e., more accurate performances) lead to larger positive reward-prediction errors. Notably, this effect was shown among successful trials, as only trials that landed on the target were analyzed. Additionally, RewP amplitude decreased as participants’ hit frequency increased, which is expected under a reinforcement learning framework. This follows because participants with higher accuracy expect to receive rewards more frequently, decreasing the size of their positive reward-prediction errors for successful performances.

Although the study by Frömer et al. (2016) makes important contributions to our understanding of how reinforcement learning principles map onto feedback processing in a more realistic setting and under different task demands (i.e., complex motor skill), the study did not explore the relationship between RewP and delayed retention (i.e., learning, as defined as a relatively permanent change in the capability for behavior; Kantak and Winstein, 2012; Schmidt and Lee, 2019). This limitation is consistent with most of the past research, which has focused on performance changes over short timescales (Bellebaum and Daum, 2008; Reinhart and Woodman, 2014), leaving a gap in the literature and making it unclear how reward-prediction errors relate to learning. From a reinforcement learning perspective, reward-prediction errors experienced during a training session drive acute behavior adaptation, leading to better practice performance and, consequently, better learning. However, motor learning studies have shown that performance during a training session does not necessarily correlate and, in some cases, may be inversely correlated with performance on delayed post-tests (Kantak and Winstein, 2012). For instance, the study by Lohse et al. (2020) showed that RewP explained some of the short-term dynamic changes in behavior but was not correlated with learning as indexed by performance on one-week retention and transfer tests. Notably, this study adopted a visual category learning task wherein participants received binary feedback (i.e., correct vs. incorrect) about their performance. Thus, it is still unclear whether reward-prediction errors measured via RewP are associated with learning of a complex motor skill in a more realistic setting (i.e., graded feedback that varies according to learners’ performance).

Building off past research (Frömer et al., 2016; Lohse et al., 2020), we tested reinforcement learning predictions and their underlying mechanisms in short- and long-term behavior adaptation by modeling data from an EEG experiment that included a complex motor task, graded feedback, and a delayed retention test. Specifically, we investigated the effect of single-trial performance accuracy on single-trial RewP during acquisition. According to reinforcement learning, more accurate performance is associated with more positive reward-prediction errors (Frömer et al., 2016). Thus, we predicted that, at the within-subject level, single-trial RewP would be more positive for more accurate compared to less accurate trials. Additionally, we examined the effect of participants’ average accuracy (at the between-subject level) on the RewP since Frömer et al. (2016) found that participants’ cumulative accuracy influenced RewP amplitude. Finally, we investigated whether aggregate RewP predicted learning as indexed by average performance on a 24-h retention test. A corollary prediction from reinforcement learning is that accrual of larger RewPs (more positive reward-prediction errors) during practice should result in a larger aggregate RewP and better learning. Thus, controlling for pretest, we predicted a positive correlation between aggregate RewP amplitude and average performance on the retention test.

## Methods

### Participants

Data from 134 participants (females = 100, *M_age_* = 20.72, *SD* = 1.64 years) were used in the present study. All participants were right-handed (*M*_handedness score_ = 77.30, *SD* = 27.24; Oldfield, 1971) or reported having a strong preference for using their right hand to throw, and reported not having any neuromuscular impairment that would affect performance of the experimental task. This dataset was collected during a larger, university-approved (Auburn University research protocol # 19–046 EP 1902) motor learning study (Bacelar et al., 2022). All participants gave written consent prior to Day 1 of data collection and verbal consent prior to Day 2 of data collection. Given the exploratory nature of the present study, there was no *a-priori* power calculation for these secondary analyses.^1^

### Task

**Figure 1:**
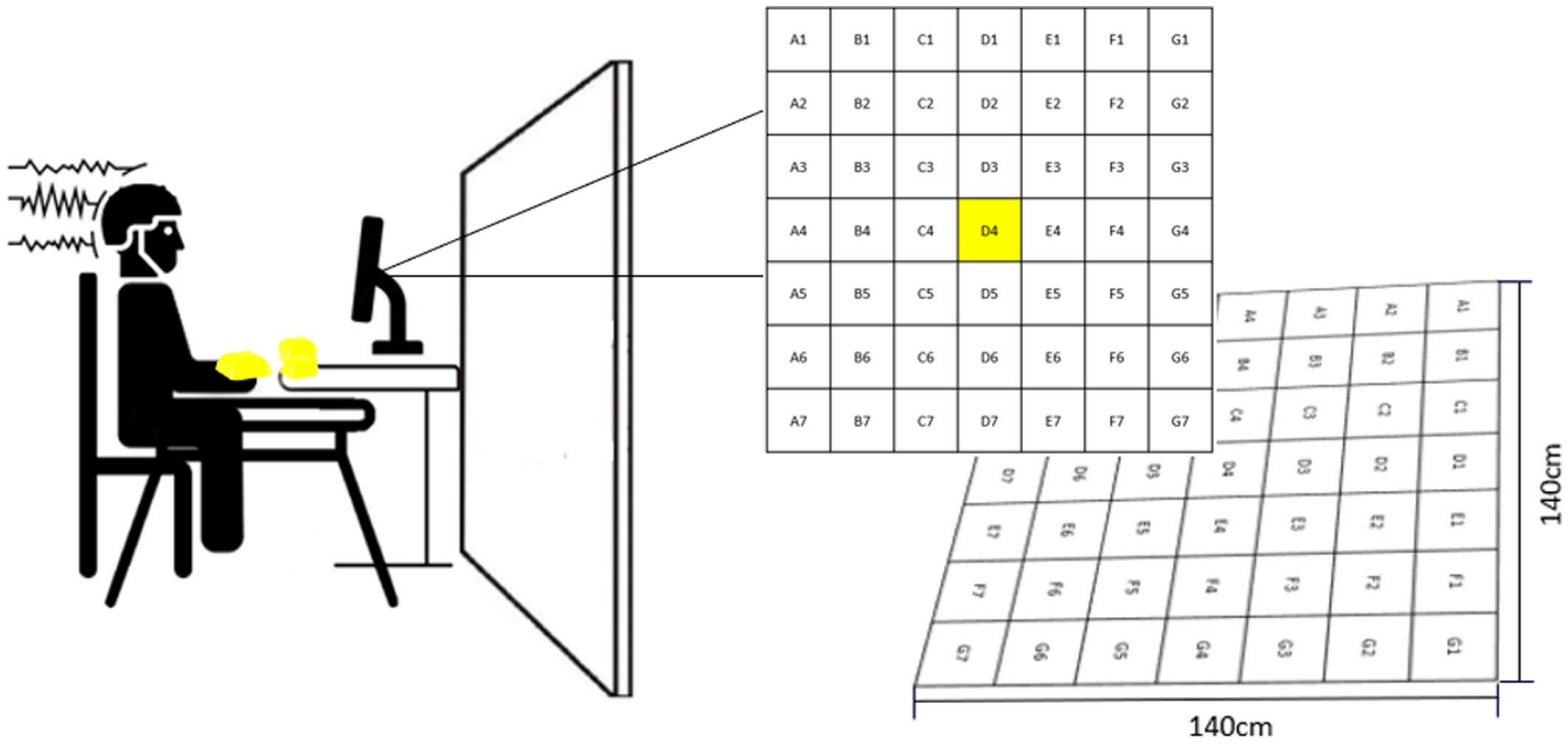
Experimental set-up. The left side of the figure shows the pasteboard which was used to occlude participants’ vision of the target. The right side of the figure illustrates how feedback was delivered throughout the experiment. This figure is a slightly modified version of Figure 1 presented in Bacelar et al. (2022). Permission to reproduce this figure has been obtained by the authors.

Participants performed a non-dominant arm bean bag tossing task. The goal of the task was to make the bean bag land as close to the center of the target as possible (i.e., D4, Figure 1). The target consisted of a grid comprising 49 equal-sized squares, each one assigned a letter and a number indicating the square position (e.g., D4: square located in the center of the target). Participants sat in front of a table located three meters away from the center of the target. The table accommodated 10 bean bags and a computer monitor used to deliver feedback in addition to serving as a support for a pasteboard used to occlude participants’ vision of the target (Figure 1). Another small table was placed next to participants’ right arm to serve as a support for a keyboard used to initiate feedback presentation. From a sitting position, participants were instructed to grasp a bean bag with their left hand pronated and toss it over the occlusion board by elevating their arm and flicking their wrist. (For more details about the task see Bacelar et al., 2022).

### Procedures

#### Acquisition phase

**Figure 2:**
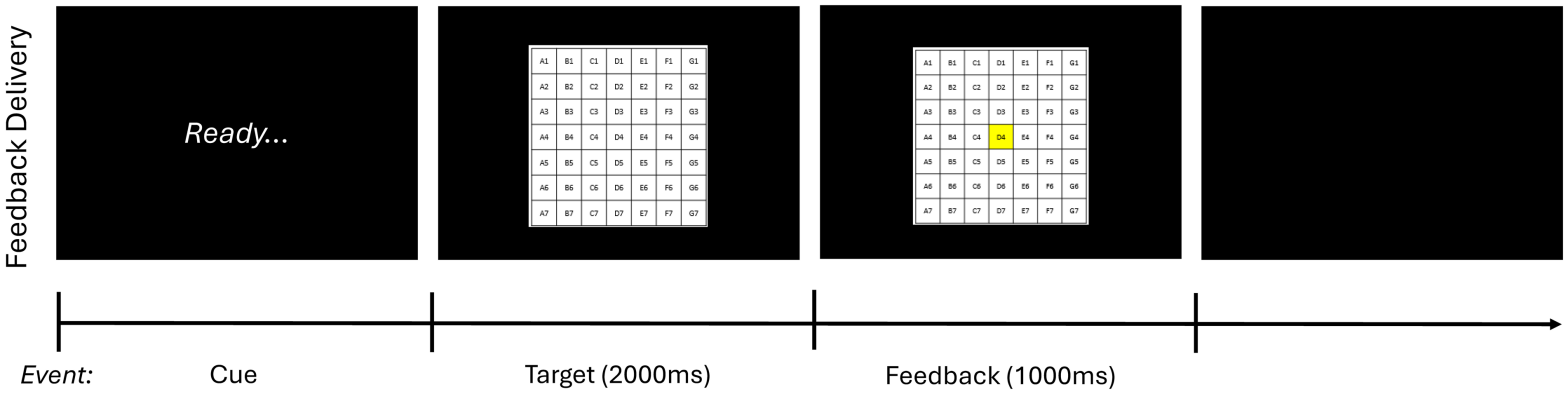
Feedback delivery sequence of events. This figure depicts how feedback was presented during the acquisition phase. On feedback trials, after throwing the bean bag and estimating their performance if applicable, participants first saw the word “Ready” on the computer screen. Next, as soon as they pressed the *enter* key on the keyboard, they saw an image of the target for 2,000 ms. Finally, for trials that landed on board, the square where the bean bag landed changed to being highlighted in yellow. This image remained on the screen for 1,000 ms. For trials that landed off board, participants saw an image of the target on the screen for 2.,000 ms followed by a red X presented for 1,000 ms. Feedback delivery was consistent across all experimental conditions with participants seeing the same sequence of events.

To determine baseline skill level, a 10-trial pretest without feedback was carried out before the acquisition phase. (Participants were allowed to see the target for 10s before initiating the pretest). Next, male and female participants were stratified and then randomly assigned based on sex to one of four experimental groups that varied according to whether feedback schedule was self-selected by the participant (self-control) or determined by a counterpart (i.e., another participant; yoked) and whether performance estimation (i.e., estimating where the bean bag landed after each trial) was required (error estimation) or not (traditional).^2^ Participants then underwent the acquisition phase, which consisted of 10 blocks of 10 trials with a 1-min break between blocks. As in the pretest, participants were allowed to see the target for 10s before initiating the acquisition phase. Feedback was presented in 5 of 10 trials per block (i.e., 50% of the time) for all participants based on their assigned experimental condition. Specifically, for feedback trials, feedback was initiated as soon as the participant pressed the “enter” key on the keyboard after being prompted by the word “ready” on the computer screen. First, participants saw an image of target on the screen for 2,000 ms and then the square where the bean bag landed changed to being highlighted in yellow, as shown in Figure 2; the latter image remained on the screen for 1,000 ms. For trials that landed off target, participants saw an image of the target on the screen for 2,000 ms followed by a red X presented for 1,000 ms.

#### Retention test

Approximately 24 h after the acquisition phase, participants returned to the laboratory to perform a retention and a transfer test. For the retention test, participants performed the same bean bag tossing task practiced on day 1, whereas for the transfer, participants were positioned farther away from the target (i.e., four instead of three meters away). Post-tests consisted of one block of 10 trials each and were carried out in counterbalanced order. Participants were allowed to see the target for 10s before initiating each post-test, but no feedback was presented during the post-test. In the present study, we focused on the results from the retention test only as this test more closely aligns with our targeted mechanism. Specifically, positive reward-prediction errors are associated with the release of dopamine (Schultz, 2017), which has been shown to modulate memory consolidation (Iemolo et al., 2015). Thus, we reasoned that results from the retention test would better represent the consolidation of the exact version of the previously acquired motor memory.

### EEG recording

EEG was recorded during the acquisition phase from 14 scalp electrodes using a 64-channel BrainVision actiCAP system (Brain Products GmbH, Munich, Germany) labeled in accord with an extended 10–20 international system (Oostenveld and Praamstra, 2001). The left earlobe served as the online reference and the FPz electrode site served as the common ground. Electrode impedances were maintained below 25kΩ throughout the experiment. A high-pass filter set at 0.016 Hz was applied and the sampling rate was set at 250 Hz. A BrainAmp DC amplifier (Brain Products GmbH) linked to BrainVision Recorder software (Brain Products GmbH) was used to amplify and digitize the EEG signal. To minimize possible EEG noise that might differ between participants, we provided EEG-specific instructions prior to initiating the recording during data collection. The instructions highlighted the need to remain still and fixate on the computer monitor when receiving augmented feedback.

### EEG processing

EEG data processing was conducted with BrainVision Analyzer 2.2 software (Brain Products GmhB). First, raw data was visually inspected and malfunctioning electrodes were interpolated. Next, data were re-referenced to the average of both left and right ears. A 1–40 Hz band-pass filter with 4^th^ order roll-offs and a 60 Hz notch filter was applied to the re-referenced data in preparation for the independent component analysis (ICA) step. Non-stereotypical artifacts were then marked throughout blocks 4 and 5, excluding the rest period between these blocks. This interval was chosen as it minimizes the presence of non-stereotypical artifacts that are either due to the participant’s adjustment to the task (i.e., earlier blocks) or tiredness (i.e., toward the end of practice). After this step, an ICA was conducted to identify components representing stereotypical artifacts (e.g., saccades and blinks), which were subsequently removed from the *unfiltered* data. Finally, the cleaned data were filtered using an infinite impulse response band-pass filter between 0.1 and 30 Hz with 4th order roll-offs and a 60 Hz notch filter.

### Measures

#### Psychophysiological measures

##### Single-trial RewP

First, filtered EEG data were segmented into epochs beginning 200 ms before and ending 800 ms after feedback stimulus onset (square highlighted in yellow or red X). To determine each participant’s RewP time-window, during the segmentation step, approximately 20 of a participants’ best trials were selected (i.e., 20 trials closest to the center of the target; Marco-Pallares et al., 2011). Some participants ended up with more than 20 trials as trials that ended in the same square of the grid counted as equally accurate, given the way that feedback was provided to participants. We also carried out trial selection in a stepwise manner. For example, trials that landed on the innermost square, D4, were included first. If the number of trials included did not add up to a minimum of 20, all trials that landed on the second group of squares surrounding the center of the target (i.e., trials landing on C3, C4, C5, D3, D5, E3, E4, and E5) were included next. This process continued until at least 20 trials were selected. After segmentation, epochs were baseline corrected from −200 ms to 0 ms. Next, consistent with the artifact rejection approach used in past studies (e.g., Meadows et al., 2016; Parma et al., 2023), epochs were automatically rejected if they contained a change of more than 50 μV from one data point to the next, a change of 100 μV or greater within a moving 200-ms window, or a change of less than 0.5 μV within a moving 200-ms window in any of the midline electrodes (Fz, FCz, Cz, and Pz). Then, to determine the time window for RewP quantification, epochs were averaged. Considering that RewP peak latency may vary across individuals, each participant’s RewP time window was adapted based on the participant’s RewP peak latency at the electrode FCz (Clayson et al., 2013). The most positive deflection within the 230–350 ms time window that exhibited a frontocentral scalp distribution was recorded (Krigolson, 2018). If no component exhibited a frontocentral scalp distribution, the most positive deflection within the 230–350 ms time window was recorded. After determining the RewP peak for each participant, data were re-segmented to include all feedback trials (i.e., 50 feedback trials). The next steps included baseline correction and epoch automatic artifact rejection following the specifications described above. The first author also visually inspected all 50 epochs and manually removed one trial that exhibited marked artifacts but was not removed in the automatic rejection step. Next, a 40-ms time window was centered on each participant’s previously recorded peak amplitude at FCz, Fz, and Cz on *each epoch*, and then mean amplitude in this time window for these electrodes was computed. The adaptive mean amplitude extraction method was used as area-based extraction methods are less sensitive to noise compared to peak-based methods (Clayson et al., 2013; Luck, 2014). Finally, we averaged across FCz, Fz, and Cz to obtain the single-trial RewP for each feedback trial.

##### Aggregate RewP

Aggregate RewP was obtained by averaging across all single-trial RewP trials for each participant.

#### Behavioral measures

##### Single-trial and average radial error

Radial error (RE) is a measure of accuracy for two-dimensional performance tasks (Hancock et al., 1995). The formula to obtain RE is as follows: (x^2^ + y^2^)^1/2^, where X and Y correspond to the magnitude of the error along the x- and y-axis, respectively. In the present study, we computed RE on a trial-by-trial basis for the acquisition phase (here referred to as single-trial RE) and as an aggregate measure for pretest, acquisition phase, and retention test (here referred to as average RE).

### Data analysis

Of the total number of single-trial RewPs (*N* = 6,700), 2.36% (*n* = 158) were lost due to either data collection issues (e.g., problems with the EEG system during data collection) and/or artifact rejection during data processing. Additionally, 215 single-trial RewPs were lost after extreme value removal (details to follow), so the final single-trial RewP count consisted of 6,327 observations. All participants included in the main analyses had at least 20 single-trial RewPs (Marco-Pallares et al., 2011). Prior to statistical analyses, we visually inspected the distribution of errors along the x- and y-axis, which led to the identification of extreme values in the y-axis. To mitigate the influence of these extreme cases on other subsequent variables (e.g., RE is computed from x- and y-axis values) and the models, we excluded errors equal to or greater than 140 cm in both directions since errors of that magnitude imply that the participant missed the center of the target by more than the length/width of the target (140 cm x 140 cm). Exclusion of extreme values in both x- and y-axis led to the loss of 577 data points (4.31% of the data; 577 of 13,400 data points).

All analyses were conducted in R (cran.r-project.org, version 4.4.0) using the following packages: *tidyverse* (version 2.0.0; Wickham et al., 2019), *car* (version 3.1.2; Fox and Weisberg, 2019), *lme4* (version 1.1.35.3; Bates et al., 2015), *lmerTest* (version 3.1.3; Kuznetsova et al., 2017), and *influence.ME* (version 0.9.9; Nieuwenhuis et al., 2012). Data visualization was carried out using the following packages: *ggplot2* (version 3.5.1, Wickham, 2016), *gridExtra* (version 2.3; Auguie and Antonov, 2017), *png* (version 0.1.8; Urbanek, 2022), and *cowplot* (version 1.1.3; Wilke et al., 2024). Alpha level was set at 0.05. For each model, residual plots were created, and residual diagnostics were computed to assess model assumptions. Data and code used to run the analyses are freely available on the project’s OSF repository.

#### Single-trial RewP, single-trial RE, and average RE models

As a first step prior to data modeling, we computed and transformed variables of interest. Specifically, we first mean-centered each participant’s single-trial RE on their average radial error to transform this variable into a within-subject measure (in contrast to average RE which served as a between-subject variable). Next, all the categorical variables were contrast-coded (group membership: self-control and error estimation) using orthogonal contrast coding with a one-unit difference (i.e., −0.5, +0.5) between levels, and average RE, treated as a continuous variable, was mean-centered. Finally, we created the quadratic and cubic terms for single-trial RE (henceforth referred to as single-trial RE^2^ and single-trial RE^3^, respectively).

Once all necessary variables were computed, we conducted model comparisons based on ANOVA ratio tests to determine whether the relationship between single-trial RE and single-trial RewP would be better captured using linear, quadratic, or cubic predictors, controlling for average RE. Terms were added to the model in order of increasing polynomial complexity, with fixed effects added before random effects. Model fit was assessed using a combination of fit indices (Akaike Information Criterion, AIC, and Bayesian Information Criterion, BIC). After a series of model comparisons, the regression model with the best fit included average RE, single-trial RE, single-trial RE^2^, and single-trial RE^3^ as fixed-effects and participant, single-trial RE, and single-trial RE^2^ as random-effects. Specifically, this model showed a reduction in AIC (≥ 2 points) and a slight increase in the BIC (5 points) compared to a model without a cubic fixed effect. The addition of a cubic random effect for single-trial RE led to a small reduction in AIC (2 points), but a large increase in the BIC (26 points). Thus, we found some evidence for the inclusion of a cubic effect (it led to reductions in the AIC), but that evidence was mixed (it led to increases in the more conservative BIC). Given that previous research has also found cubic relationships between performance accuracy and RewP (Frömer et al., 2016), we ultimately decided in favor of the simpler cubic model, with only a cubic fixed effect.

Finally, to assess the relationship between single-trial RewP and single-trial RE and whether this relationship was moderated by participants’ average performance during acquisition (i.e., average RE), we ran a mixed-effects regression model with fixed effects of group membership (self-control, error estimation, and their interaction) plus an interaction between participant’s average RE and their single-trial RE to the best fitting model above. Single-trial RewP served as the dependent variable in the model.

#### Aggregate RewP and retention model

To assess whether aggregate RewP predicted average RE in the retention test, we ran a general linear regression model with average RE in retention serving as the dependent variable and aggregate RewP as the predictor, controlling for pretest and group membership. All continuous variables were mean-centered, and the categorical variables were contrast coded using orthogonal contrast coding with a one-unit difference (i.e., −0.5, +0.5) between levels.

## Results

**Figure 3:**
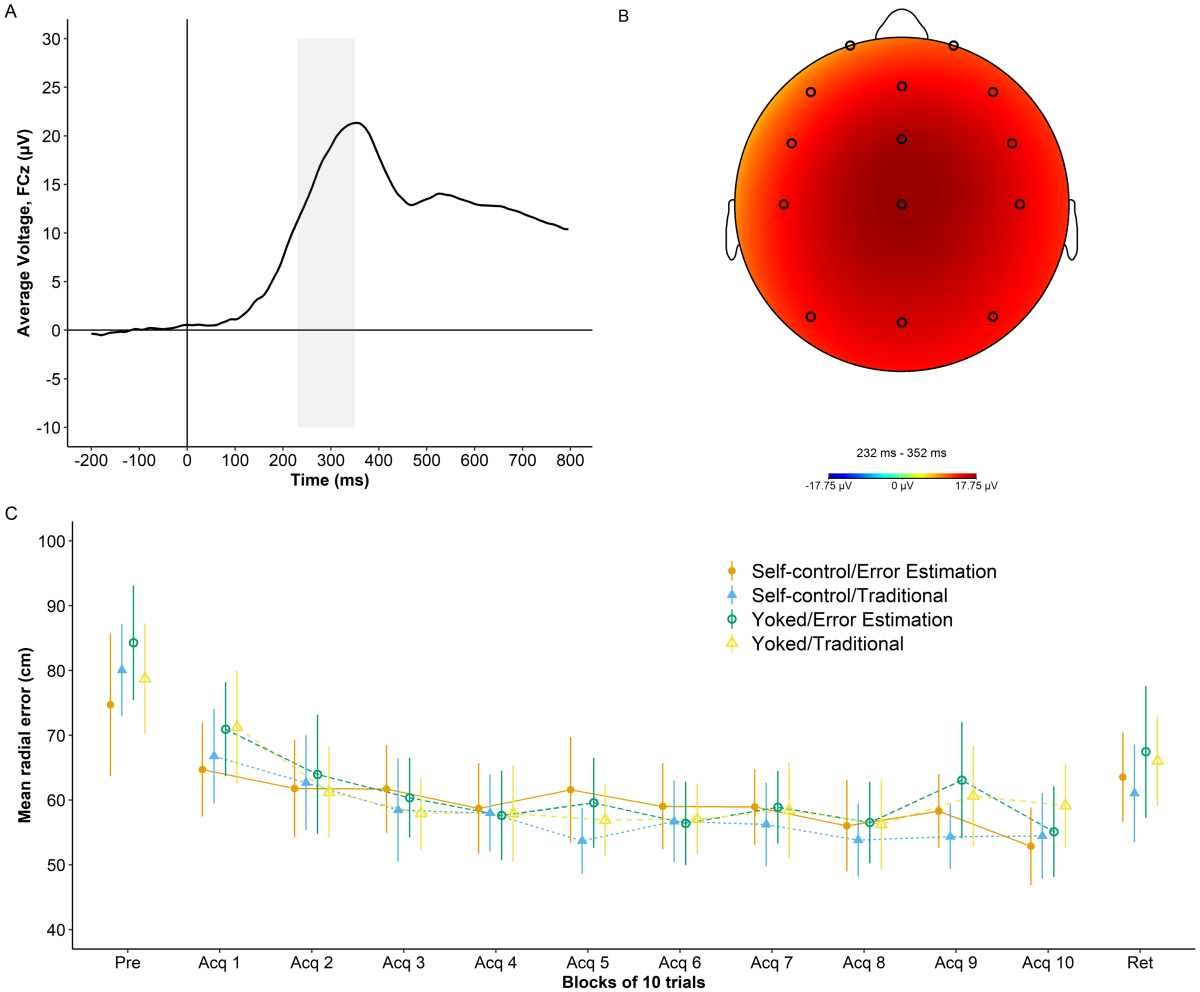
Psychophysiological and behavior data. (A) Grand average waveform for the RewP time-locked to the onset of augmented feedback (time 0) at electrode FCz. Shaded area represents the RewP time window (230 ms-350 ms). (B) Topography of the RewP averaged across trials and training conditions. (C) Radial error in cm (lower numbers indicate better performance) as a function of group and study phase (pretest, acquisition, and retention). Error bars represent 95% CIs. For reference, the behavioral analyses presented in Bacelar et al. (2022) showed non-significant effects of self-control, error estimation, and Self-control x Error Estimation (*ps* ≥ 0.805) for the dependent variable average radial error during acquisition at the group level. Similar results were found for average radial error during retention with non-significant effects of self-control, error estimation, and Self-control x Error Estimation (*ps* ≥ 0.255).

An overview of RewP grand average at electrode FCZ, RewP topography, and performance accuracy across study phases is presented in Figure 3.

### Single-trial RewP, single-trial RE, and average RE

**Figure 4:**
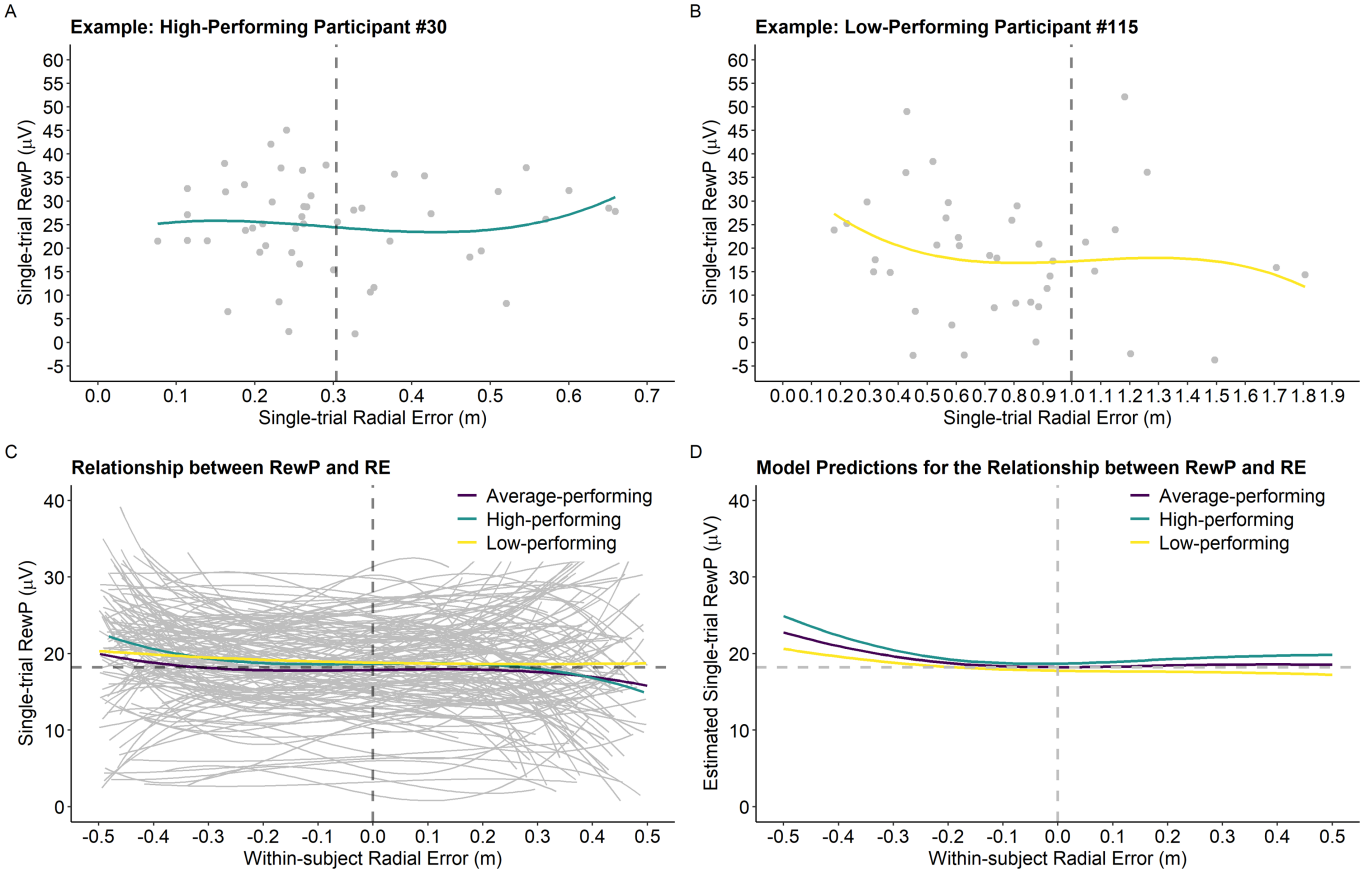
Single-trial RewP as a function of single-trial RE and participants’ performance level. (A) Example of the relationship between the variables of interest for a high-performing participant. The vertical dashed line represents the participant’s average RE (0.31 m). (B) Example of the relationship between the variables of interest for a low-performing participant. The vertical dashed line represents the participant’s average RE (1.00 m). (C) Graph depicting the relationship between single-trial RewP and single-trial RE as a function of average RE. For visualization purposes only, we created a categorical variable called *performance level* based on the continuous variable *average RE.* Specifically, participants were classified into three distinct performance levels by dividing average RE into tertiles. Thus, the high-performing, average performing, and low-performing categories depicted in the graph (panels C,D) correspond to the bottom, middle, and top tertiles of the average RE distribution, respectively (High-performing: 0.3 m-0.51 m, *n* = 45; Average-performing: 0.52 m-0.62 m, *n* = 45; and Low-performing: 0.63 m-1.23 m, *n* = 44). Notably, Average RE was treated as a continuous variable in all statistical models. The horizontal dashed line represents the intercept predicted by the model (average RewP; 18.21 μV) and the vertical dashed line was centered on zero as it represents participants’ average trial accuracy. (D) Model predictions for the relationship between single-trial RewP and single-trial RE as a function of average RE (in the graph represented by the variable *performance level*).

Results of the analysis investigating the relationship between single-trial RewP, single-trial RE, and average RE are presented in Table 1. The model revealed a main effect of single-trial RE^2^ (*p* < 0.001) and single-trial RE^3^ (*p* < 0.001), which were superseded by Average RE x Single-trial RE^2^ (*p* = 0.028) and Average RE x Single-trial RE^3^ (*p* = 0.039) interactions. Figure 4D depicts the model’s predictions for single-trial RewP as a function of single-trial RE and average RE. Overall, the cubic relationship between single-trial RewP and single-trial RE indicates that single-trial RewP was more responsive to errors that deviated considerably from the participants’ average error. Specifically, single-trial RewP amplitude was largest following participants’ best trials (i.e., smallest single-trial RE; see left part of the graph in Figures 4C,D) and smallest following participants’ worst trials (i.e., largest single-trial RE; see right part of the graph in Figures 4C,D). Moreover, the moderating effect of average RE indicates that the RewP-accuracy relationship depended on participants’ average performance. According to the model’s predictions, high-performing participants showed larger RewPs for trials that were considerably better than their average error and smaller RewPs for trials that were worse than the participant’s average error. On the other hand, for low-performing participants, the RewP responses to better-than-expected and worse-than-expected outcomes were not as distinct.

**Table 1:** Random and fixed effects for the analysis of the relationship between single-trial RewP, single-trial RE, and average RE.

Random Effects					
Group	Effect	Variance	SD	Corr	
Participant	Intercept	65.20	8.08		
	Single-trial RE	40.80	6.39	0.08	
	Single-trial RE^2^	190.60	13.81	0.04	0.34
Residual		141.40	11.89		

Fixed Effects				
Effects	*β*	95% CI	*t*-value	*p*-value
Intercept	18.21	[16.78; 19.64]	24.96	<0.001***
Self-control	−2.00	[−4.83; 0.82]	−1.39	0.167
Error estimation	−2.00	[−4.82; 0.82]	−1.39	0.168
Average RE	−3.08	[−12.63; 6.47]	−0.63	0.528
Single-trial RE	−0.12	[−2.46; 2.21]	−0.10	0.917
Single-trial RE^2^	9.73	[5.13; 14.33]	4.15	<0.001***
Single-trial RE^3^	−16.89	[−26.45; −7.34]	−3.47	<0.001***
Self-control x error estimation	1.28	[−4.36; 6.92]	0.44	0.658
Average RE x single-trial RE	−7.39	[−22.38; 7.61]	−0.97	0.335
Average RE x single-trial RE^2^	−33.43	[−62.95; −3.91]	−2.22	0.028*
Average RE x single-trial RE^3^	53.49	[2.79; 104.18]	2.07	0.039*

Number of observations: 6327, groups: Participants: 134 (One of the participants included in this analysis ended up losing 50 out of 100 radial error trials due to an iPad malfunction. To determine whether this participant showed any sign of substantial influence on the model that would justify their exclusion from the analysis, we computed Cook’s distance statistics and DFBETAS. Upon analysis of these measures, it was determined that this participant did not significantly influence the model, so they were kept in the analysis. For the interested reader, the R code allows a sensitivity analysis without this participant to be conducted. In sum, the results do not change after the participant’s exclusion from the dataset). Self-control was coded as: Self-control = −0.5; yoked = 0.5. Error Estimation was coded as: Error estimation = −0.5; traditional = 0.5. The marginal R^2^ value was 0.02 and the conditional R^2^ value was 0.35. These R^2^ values were computed using the MuMIn package (Bartoń, 2023), which uses the formulas presented in Nakagawa and Schielzeth (2013).

### Aggregate RewP and retention

Results of the analysis of the relationship between aggregate RewP and retention showed no significant main effect of aggregate RewP (*p* = 0.273), after controlling for pretest performance and group membership. Thus, RewP did not predict learning.

## Discussion

Different learning models are used to explain the process by which humans acquire motor skills. Error-based learning explains motor adaptation on the account of sensory-prediction errors, where the learner relies on a forward model of the action to predict the sensory consequences of said action. Based on the discrepancy between anticipated and actual sensory outcome, the learner computes a sensory-prediction error that is used to update the internal representation of the skill and implement future motor adjustments (Haith and Krakauer, 2013b). Use-dependent learning, on the other hand, does not seem to depend on the existence of an internal model; instead, movement refinement is explained through mere motor action repetition (Classen et al., 1998; Diedrichsen et al., 2010). Finally, reinforcement learning explains skill acquisition as the result of successful actions being reinforced via reward-prediction errors. According to this model, learners use outcome-based information to compute the difference between expected and actual reward and, based on the difference, implement behavioral adjustments to increase the likelihood of receiving rewards in the future. Importantly, the concept of rewards in reinforcement learning models is broad, ranging from financial incentives (e.g., Abe et al., 2011) to experiences of success in a task (e.g., Wulf and Lewthwaite, 2016). Thus, anything that has some utility can function as a reward (Lohse et al., 2019). Although motor skill acquisition likely depends on the interaction between different learning models, in the present study we tailored our research questions, predictions, and analyses to focus on reinforcement learning, which has been proposed as one of the dominant learning processes when it comes to skill tasks (Haith and Krakauer, 2013b; Lohse et al., 2019). Specifically, we implemented mixed-effects and linear regression models to explore a 134-participant dataset consisting of learners’ feedback-evoked EEG activity (i.e., RewP) as well as their short- and long-term motor performance to investigate reinforcement learning predictions and mechanisms in a motor learning context. As primary goals, we investigated the effect of trial accuracy on reward-prediction errors as indexed by the RewP and whether this relationship was moderated by participants’ average performance. We also examined whether aggregate RewP predicted motor learning as indexed by performance in a delayed (~24 h) retention test.

Results revealed that the relationship between single-trial RewP and single-trial RE is more complicated than originally predicted. Specifically, performance on the current trial affected the RewP in a non-linear manner, and this relationship was moderated by participants’ average performance during acquisition. As illustrated in Figure 4, for a high performer (lower average RE), single-trial performances that were better than the average error for that participant resulted in very large RewPs, whereas single-trial performances that were worse than the average error resulted in smaller RewPs, with these effects being larger for extreme values (i.e., farther away from the average performance). Following reinforcement learning and the changes observed during the motor skill acquisition process (Lohse et al., 2019), high-performing participants may have leveraged the outcome-based feedback to supplement the sensory-feedback available and develop a stronger representation of the skill throughout the practice phase, which resulted in more stable predictions about their outcomes (i.e., they were able to make predictions based on their global performance). Thus, when their expectations were violated by a much better-than-average or much worse-than-average outcome, this resulted in a larger positive/negative reward-prediction error (i.e., larger/smaller RewP, respectively). In contrast, a low-performing participant was less sensitive to these violations in prediction errors, probably because their average performance was more variable (i.e., noisier). Descriptively, the variance of single-trial RE for participants whose average RE was one standard deviation above the mean was 0.29 m as opposed to a variance of 0.05 m observed for participants one standard deviation below the mean. Thus, a low-performing participant, likely influenced by their variable performance, had yet to develop a reliable representation of the skill, which resulted in performance expectations that were very uncertain (e.g., the participant was not able to accurately distinguish a good from a bad performance based on their average performance). This is illustrated by the slope of the curve representing the relationship between single-trial RewP and single-trial RE in Figure 4, where the amplitude of single-trial RewPs for better-than-average and worse-than-average performances is attenuated for low-performing participants.

Parallels can be drawn between the present and past research findings. For instance, Frömer et al. (2016) also found a cubic relationship between motor accuracy and RewP amplitude in response to graded feedback. Additionally, RewP amplitude was influenced by participants’ cumulative hit frequency rate such that a high-frequency rate during practice resulted in overall smaller RewP amplitudes, especially after a hit trial. This suggests that as learners got better at the task, their accumulated successful hits led them to predict successful outcomes more frequently, lowering their reward-prediction errors. Importantly, this RewP amplitude modulation by both accuracy and hit frequency rate was shown within successful trials as only trials that hit the target were analyzed. In the present study we extend these results by showing how trial accuracy and average accuracy affected the RewP across successful and unsuccessful trials as both on- and off-target trials were analyzed. This is important considering that negative feedback is also informative (Gershman, 2015), and can contribute to the update of outcome expectations and be used to guide future behavior adaptations (e.g., explore different actions that may lead to a different outcome). Along the same lines, an interesting avenue for future research is to investigate how graded feedback processing across the entire reward magnitude range (from successful to unsuccessful trials) affects future behavior adaptations. For instance, in their perceptual category learning experiment, Lohse et al. (2020) found that a larger RewP amplitude (possibly representing a violation of outcome expectation) was associated with a greater probability of changing the response the next time a stimulus from the same category was presented. These trial-by-trial adaptive dynamics are likely more complex in motor skill acquisition as there may not always be a correspondence between one’s capability to select the correct action and execute the appropriate motor response (Bacelar et al., 2020; McDougle et al., 2016), but any insights into these dynamics will lead to a better understanding of how graded feedback is used to guide performance adjustments during practice.

Another interesting finding from our study was the moderator effect of between-subject average performance on the RewP-accuracy relationship. Although RewP modulation by contextual factors has been shown in more controlled experiments (Holroyd et al., 2004), in the present study this modulation occurred in a more naturalistic setting where the contextual factor (average performance) was a direct consequence of participants’ behavior adjustments implemented during practice. Overall, we found that high-performing participants were more sensitive to violations in prediction errors. From a motor learning standpoint, understanding how learners at different skill levels process feedback can offer important insights into the optimal implementation of reinforcement learning to enhance skill acquisition. For instance, it is possible that learners’ average performance level might influence the degree to which they find the response-based feedback informative. For a high-performing learner, providing graded outcome feedback may favor performance improvements as this learner’s stronger internal representation of the skill and better error detection capabilities might allow them to extract useful information from the type of feedback provided. On the other hand, a low-performing learner having to rely on less stable prediction errors may find it challenging to extract useful information from the same type of feedback. Thus, depending on the learner’s skill level, a practitioner or a coach might decide to rely on different error correction strategies to better support their learners. For example, they might use knowledge of *results*, a type of augmented feedback that informs the learner about the success of an action in reference to a goal (e.g., you missed the target by 32 cm; Schmidt and Lee, 2019) to assist high-performing learners who can rely on a stronger internal model to compute reward-prediction errors based on the outcome feedback, and select and execute movements that maximize the reward-prediction errors. Alternately, the practitioner/coach might use knowledge of *performance*, a type of augmented feedback that provides information about the movement pattern and, therefore, can be used to guide the learner toward the appropriate movement solution (e.g., fully extend your arms after you hit the ball to ensure a powerful swing; Schmidt and Lee, 2019) to assist low-performing learners who may need more guidance due to their poorly developed internal model. In line with the challenge-point framework (Guadagnoli and Lee, 2004), another strategy that an instructor might consider is adjusting the difficulty of the task at hand based on how challenging the task is for the learner, which is directly associated with the learner’s current skill level, and also the goals of practice (e.g., practice-to-learn versus practice-to-maintain; Hodges and Lohse, 2022). According to this framework, learning is enhanced when individuals practice within their range of optimal difficulty. Drawing parallels with the results of the present study, it is possible that our task was within this range of optimal challenge for our high-performing participants, but outside this range (too functionally difficult) for our low-performing participants. Thus, if the goal of practice is to promote learning, an instructor working with a low-performing learner should consider simplifying the task at hand (e.g., by adjusting task-specific constraints; Brocken et al., 2020) so that this learner has the opportunity to practice under a level of challenge that is optimal for them. Reducing the difficulty of the task in this scenario satisfies the concept of desirable difficulties to promote skill learning (Bjork and Bjork, 2020; Christiansen et al., 2020; Guadagnoli and Lee, 2004; Hodges and Lohse, 2022) and, at the same time, might lead to reduced performance variability, allowing the learner to create a better representation of their average performance, which might lead to more stable prediction errors and better response-based feedback interpretations.

Finally, contrary to our prediction, the analysis of the relationship between aggregate RewP and motor learning showed that aggregate RewP did not predict performance on the retention test. Similar results were found in a categorical learning study by Lohse et al. (2020) where aggregate RewP amplitude did not predict performance on one-week retention and transfer tests. In another study in the motor learning domain, RewP was associated with practice performance but not learning (Grand et al., 2017). This lack of association between RewP and retention observed in the present and past studies questions whether the RewP is a reliable marker of motor skill learning. One possibility for this lack of an association is that the RewP may be epiphenomenal, reflecting a violation of reward expectation that is a byproduct of changes that have already taken place (i.e., internal model update), rather than the reward mechanism that *causes* behavioral adaptation. The potentially epiphenomenal nature of the RewP is consistent with past work showing that participants are more likely to change their responses following a large RewP, controlling response accuracy (e.g., Lohse et al., 2020; Philiastides et al., 2010). That is, the RewP is more reflective of updated internal models and thus participants’ predictions, than it is reward.

Alternatively, the RewP may reflect reward-prediction errors that are driving motor adaptation during practice, but successful learning (as shown on delayed retention tests) further requires three interdependent processes: encoding, consolidation, and retrieval (Kantak and Winstein, 2012). Thus, the RewP might be associated with behavioral adjustments that occur during practice and that are important for encoding relevant information (e.g., making associations between movement and movement outcome), but this adaptive behavior might not be consolidated (e.g., contextual interference effect; Magill and Hall, 1990). In line with this idea, the RewP might influence other learning mechanisms such as reasoning inference (Tsay et al., 2024). Under this perspective, the RewP signal might assist learners in understanding how their actions map onto outcomes either through explicit or implicit strategies (for more details, see the 3R framework for motor learning proposed by Tsay et al., 2024). In our bean bag tossing task, this means learning the association between the arm swing force as well as the wrist angle prior to object release and the bean bag’s trajectory and how that affects the object’s final landing position in reference to the center of the target (i.e., main goal). An exciting avenue for future research could explore the relationship between outcome-based feedback and trial-by-trial error adjustments during acquisition (e.g., Lohse et al., 2024) and whether that relationship is moderated by event-related potentials (e.g., RewP, P300; Palidis et al., 2019) and/or oscillatory neural markers (e.g., theta lateralization; Rampp et al., 2022). Taken together, the present and past studies draw attention to the importance of examining the RewP-behavior adaptation relationship over different timescales, especially in studies focused on uncovering the basis of the learning process, as there may not be a direct correspondence between RewP and behavior adaptation over the short and long term. Our findings also strengthen the notion that the motor skill acquisition process is complex and multifaceted, and that a more complete explanation of its neural underpinnings requires an approach that considers different learning models, including but not limited to reinforcement learning, error-based learning, and/or use-dependent learning (Kantak and Winstein, 2012). For instance, research has shown that error-based learning and use-dependent learning can work together simultaneously to promote motor adaptation (Diedrichsen et al., 2010). Along the same lines, use-dependent learning has been shown to be enhanced when relevant outcome-based feedback is present and reinforcement learning is induced (Mawase et al., 2017). Thus, consideration of the interplay between different learning models would promote a more comprehensive understanding of how humans acquire motor skills.

While the present study has many strengths across conceptualization (i.e., use of theory-based predictions), experimental design (e.g., use of graded, realistic feedback, delayed retention test, and large dataset) and data analysis (e.g., implementation of mixed-effects regression modeling), some limitations are worth discussing. For one, the choice of task might raise the question of whether our results can be generalized to tasks that have different requirements or constraints, for example when learners have to perform under higher cognitive load (e.g., dual-tasking), the availability of augmented feedback is limited, and/or the task itself imposes greater motor demands. Regarding dual-task performance, it directly affects attention allocation and working memory (Schmidt and Lee, 2019), which might affect one’s ability to learn from rewards. Indeed, past research has shown that RewP amplitude is reduced when cognitive load increases, suggesting that the reward system’s capacity to process rewards is reduced in these situations (Krigolson et al., 2015), and that reinforcement learning might be limited in dual-task conditions (Holland et al., 2018). Regarding tasks where augmented feedback is limited or not available, learners may rely on intrinsic feedback (e.g., proprioceptive and/or visual information) to compute sensory-prediction errors that engage error-based learning and/or reward-prediction errors that engage reinforcement learning. Research has shown that differences in the availability and/or quality of visual feedback might affect the extent to which learners rely more on sensory- or reward-predictions errors (Izawa and Shadmehr, 2011). Finally, for very complex tasks with greater variability in motor commands, learners relying on outcome-based feedback might face the credit-assignment problem (Dam et al., 2013; Lohse et al., 2019). Specifically, they may not be able to determine which component of the generated action led to their better-than- or worse-than-expected outcome, rendering reward-prediction errors less useful for adapting behavior in this context. These considerations highlight the importance of studying feedback processing across a variety of tasks to promote result generalizability. While our bean bag tossing task allowed us to successfully quantify feedback processing as it relates to basic aspects of motor skill acquisition, it might be considered too simplistic in the realm of ecologically-valid motor tasks. Moreover, ecological validity was further reduced in our experiment by controlling when participants received augmented feedback about the outcome of their action. This methodological decision was made so that an event-maker time-locked to feedback onset could be reliably recorded. Otherwise, it is difficult to determine when participants begin processing feedback. It is also worth mentioning that we analyzed data from a homogenous sample consisting of college-age, healthy young adults. Thus, our convenience sample may limit the generalizability of our findings to other populations. This is an important limitation as reward processing has been shown to change with aging (Vink et al., 2015). Future research should consider investigating the relationship between feedback processing and behavioral adjustments in more ecologically-valid tasks and across different populations to enhance our understanding of the processes underlying motor skill acquisition. Finally, while theoretical and methodological considerations were taken into account to build our mixed-effects models, we acknowledge that there may be more complex and sophisticated ways to capture the dynamics between RewP, trial accuracy, and average performance. For instance, RewP amplitude has been shown to change as a function of practice (Williams et al., 2018), presumably due to rewards becoming more likely (Frömer et al., 2016) and/or the development of a stronger internal model representation (Lohse et al., 2020). In the present study, we attempted to capture the effect of practice on the RewP-accuracy dynamic by creating a within-subject accuracy variable (single-trial RE) and a between-subject accuracy variable (average RE) and adding these variables as predictors in the model. Other researchers have taken different approaches such as creating a running average variable representing the mean error up until the current trial (Frömer et al., 2016). Future research interested in disentangling these complex dynamics may consider accounting for the effect of practice on the RewP by more explicitly modeling time, for example by adding trial number to the fixed and/or random components of the model.

In summary, the present study showed how the relationship between RewP and trial accuracy unfolded in a non-linear manner and was moderated by participants’ average performance, which can be explained by reinforcement learning predictions. Importantly, these complex dynamics involving graded feedback, accuracy, and RewP amplitude were shown across successful (on-target) and unsuccessful (off-target) trials, furthering our understanding of graded feedback processing in motor skill acquisition. Moreover, even though our results support the use of reinforcement learning predictions to explain variations in reward-prediction errors as a function of trial and average accuracy, this association was restricted to short-term performance as there was no evidence of the relationship between aggregate RewP and motor learning. Nonetheless, we argue that reinforcement learning as a mode of learning may still be an important model to understand behavior adaptation and motor memory consolidation. At least at the behavior level, recent evidence suggests that reinforcement learning approaches may lead to better motor skill retention compared to other modes of learning (Magelssen et al., 2024; Truong et al., 2023). Thus, we encourage future investigations into the application of reinforcement learning theory and its neural mechanisms to explain motor skill learning.

## Data Availability

The datasets presented in this study can be found in online repositories. The names of the repository/repositories and accession number(s) can be found at: https://osf.io/r3qm2/?view_only=3cb3b2c773694ac2800e14edbbf9e698.

## Footnotes

^1^An *a-priori* power analysis was conducted to estimate the sample size needed to test the main hypothesis of the behavioral study (detailed parameters to replicate this power analysis can be found in Bacelar et al., 2022). Based on this formal power analysis, we first aimed to collect EEG data from 200 participants. However, due to the COVID-19 global pandemic, EEG data collection was stopped to reduce the duration of the experiment and minimize participants’ exposure to the virus. Thus, our final 134-participant dataset reflects the much-needed adjustments implemented in response to the pandemic.

^2^These experimental conditions were created to test predictions made by a motor learning theory (OPTIMAL theory; Wulf and Lewthwaite, 2016) and the results of these manipulations have been presented in another publication (Bacelar et al., 2022). We account for these manipulations in our statistical models even though they are not of primary interest in the present study.
